# Ceremonies among the Hottentots

**Published:** 1901-03

**Authors:** 


### CEREMONIES AMONG THE HOTTENTOTS.

L'INDEPENDENCE MEDICALE prints the following account of the ceremonies at birth and at puberty practiced by the Hottentots:

When the woman begins to feel labor pains, the neighbors gather in her hut, from which the husband is rigorously excluded. Parturition is generally prompt and easy. If it is difficult, the mother is given a draught of a decoction of tobacco in milk to aid delivery. As soon as the child is born, it is bathed in cow's urine and smeared over with tallow and figtree juice, then covered with an odorous powder.

The event is usually hailed with joy and celebrated by a feast for which an ox or a sheep is killed, according to the means of the parents. The birth of twins, far from displeasing the father, fills him with joy and pride. It is only in cases of extreme misery and where it is impossible for the mother to take care of two children at a time, that the Hottentots decide with great reluctance to sacrifice one of them.

The mother always nurses the child and is never separated from it even when at work. She carries it on her back supporting it on her buttocks, which are often abnormally developed. The exactions of maternity constitute a motive for the women to smoke incessantly, and to amuse their infants they early initiate them in the seductions of the pipe. When the child has no longer need of its mother, it is turned loose and allowed to exercise and develop its limbs according to its inclination. The dystrophies of childhood are rarely seen among the Hottentots.

The time of puberty is celebrated by the two sexes with grand feasting. The young people among some of the tribes are made to undergo a peculiar operation accompanied by strange, and to our eyes, disgusting ceremonies. The young girls, who are as naked as at birth, take at this time the skin apron. After being exposed for three days at the door of the hut, a young cow is killed in their honor. An unmarried relative compliments them by placing on their heads the udder of the animal in wishing for them a fecundity like that of the cow.

---

THE following officers were elected in the national societies which recently met in Baltimore: American Psychological Association—Professor Josiah Royce, president; Dr. Livingston Farrand, secretary. Society for Plant Morphology and Physiology—Dr. Edward F. Smith, president; Professrs F. C. Newcombe and L. M. Underwood, vice-presidents; Professor W. F. Young, secretary and treasurer. American Morphological Society—Professor J. S. Kingsley, president; Professor E. A. Andrews, vice-president; Professor T. H. Montgomery, Jr., secretary. American Association of Bacteriologists—Professor William H. Welch, president; Professor E. O. Jorden, vice-president; Professor H. W. Conn, secretary and treasurer.

---

THE oldest qualified physician in the world resides at Carlsbad. He is ninety-seven years of age, has been in practice for seventy-one years, and is in full possession of his mental faculties. His name is Ritter von Hochburger.

